# Signal transduction schemes in *Pseudomonas syringae*

**DOI:** 10.1016/j.csbj.2020.10.039

**Published:** 2020-11-09

**Authors:** Yingpeng Xie, Wenbao Liu, Xiaolong Shao, Weihua Zhang, Xin Deng

**Affiliations:** a Department of Biomedical Sciences, City University of Hong Kong, Kowloon Tong 999077, Hong Kong Special Administrative Region; b Shenzhen Research Institute, City University of Hong Kong, Shenzhen 518057, China; c College of Agricultural Sciences and Technology, Shandong Agriculture and Engineering University, Jinan 250100, China; d Institute of Vegetables and Flowers, Shandong Academy of Agricultural Sciences, Jinan 250100, China

**Keywords:** *Pseudomonas syringae*, Signal transduction systems

## Abstract

To cope with their continually fluctuating surroundings, pathovars of the unicellular phytopathogen *Pseudomonas syringae* have developed rapid and sophisticated signalling networks to sense extracellular stimuli, which allow them to adjust their cellular composition to survive and cause diseases in host plants. Comparative genomic analyses of *P. syringae* strains have identified various genes that encode several classes of signalling proteins, although how this bacterium directly perceives these environmental cues remains elusive. Recent work has revealed new mechanisms of a cluster of bacterial signal transduction systems that mainly include two-component systems (such as RhpRS, GacAS, CvsRS and AauRS), extracytoplasmic function sigma factors (such as HrpL and AlgU), nucleotide-based secondary messengers, methyl-accepting chemotaxis sensor proteins and several other intracellular surveillance systems. In this review, we compile a list of the signal transduction mechanisms that *P. syringae* uses to monitor and respond in a timely manner to intracellular and external conditions. Further understanding of these surveillance processes will provide new perspectives from which to combat *P. syringae* infections.

## 1 Introduction

All organisms display a remarkable ability to acclimate to their natural habitats. As simple unicellular microorganisms, bacteria have their own versatile devices for evoking appropriate cellular responses to adjust smoothly to their environments. In many pathogens, certain stimuli evoke the synthesis of diverse virulence factors to enable host invasion [1]. Since the early 1990s, signal transduction systems in bacteria have been widely studied and discussed. Two-component regulatory systems (TCSs) and methyl-accepting chemotaxis proteins (MCPs) are phosphotransferase adopted by bacteria as adaptive responses to changing environmental conditions [2], [3], [4], [5], [6]. As more mechanisms of signal transduction have been elucidated in recent decades, the cytoplasmic components used to monitor the intracellular and cell envelope conditions have been revealed [7], [8], [9]. A recent review classifies bacterial sensor proteins into six categories according to the signal transduction machinery: 1) TCSs; 2) MCPs; 3) membrane components of the sugar phosphotransferase system; 4) nucleotide-based secondary messengers and related enzymes; 5) extracytoplasmic function (ECF) sigma factors and 6) Ser/Thr/Tyr protein kinases and phosphatases [10]. Even among closely related microorganisms, different bacterial pathogens show biased distributions of sensor proteins [8]. Individualised signalling systems harness bacteria to elicit favourable responses to environmental conditions.

*Pseudomonas syringae* pathovars are widespread pathogens that infect various staple crops, thus causing huge economic losses and presenting a threat to food security worldwide [11], [12]. *P. syringae* also serves as a model strain for studying plant-pathogen interactions, microbial pathogenicity and microbial ecology [12], [13]. The impacts of *P. syringae* on both scientific and economic grounds contribute to its position as a premier plant pathogen [12]. Like many other phytopathogenic bacteria, *P. syringae* deploys its type III secretion system (T3SS) to invade host plants and cause lethal diseases [14], [15]. The expression of T3SS genes is repressed when bacteria are cultured in nutrient-rich medium such as King’s B medium (KB), but rapidly induced to high levels when grown on plants or in minimal medium (MM) [16], [17], [18]. The MM is believed to resemble the environment of plant intercellular spaces where bacteria grow [16]. The number of proteins associated with signal transduction is usually considered a criterion of a bacterium’s ability to adapt to changing surroundings [8]. According to this standard, *P. syringae* is particularly ‘smart’ when compared with other phytopathogenic bacteria. For example, the genome of *P. syringae* pv. *tomato* DC3000 strain (*Pto*DC3000, a model pathogen on *Arabidopsis* and tomato) encodes 279 signal transduction proteins, which account for 4.9% of the total of 5,608 proteins produced by this strain [8], [19]. This number is much higher than that in other plant-pathogenic bacteria, such as *Ralstonia solanacearum* (161 signal transduction proteins among all 5,116 proteins, 3.1%), *Agrobacterium tumefaciens* (163 signal transduction proteins among all 5,402 proteins, 3.0%) and *Xylella fastidiosa* (39 signal transduction proteins among all 2,832 proteins, 1.3%) [8]. The presence of these many signalling proteins in *P. syringae* suggests the high sensitivity and adaptability of the species in response to changing environmental conditions. An understanding of the signal transduction networks of *P. syringae* is essential for deciphering its pathogenicity and responses to stresses. This review focuses on a group of well-studied signalling systems, including TCSs, MCPs, ECF sigma factors, secondary messengers and other intracellular surveillance systems, which illustrate how extracellular stimuli evoke corresponding cellular responses in *P. syringae.*

## 2 TCSs

A TCS is composed of a histidine kinase (HK) and its cognate response regulator (RR) that enable the proteins to transduce external cues into intracellular signals through the transfer of phosphoryl groups [3], [20], [21]. RRs display RNA/DNA-binding, protein-binding or enzymatic activities, which modulate a wide range of cellular activities [22]. Although most HKs are membrane-bound, there are a significant number of TCSs that are soluble and present in the cytosol (HKs lack transmembrane regions).

### 2.1 Temperature-sensing TCS CorRS

**Fig. 1:**
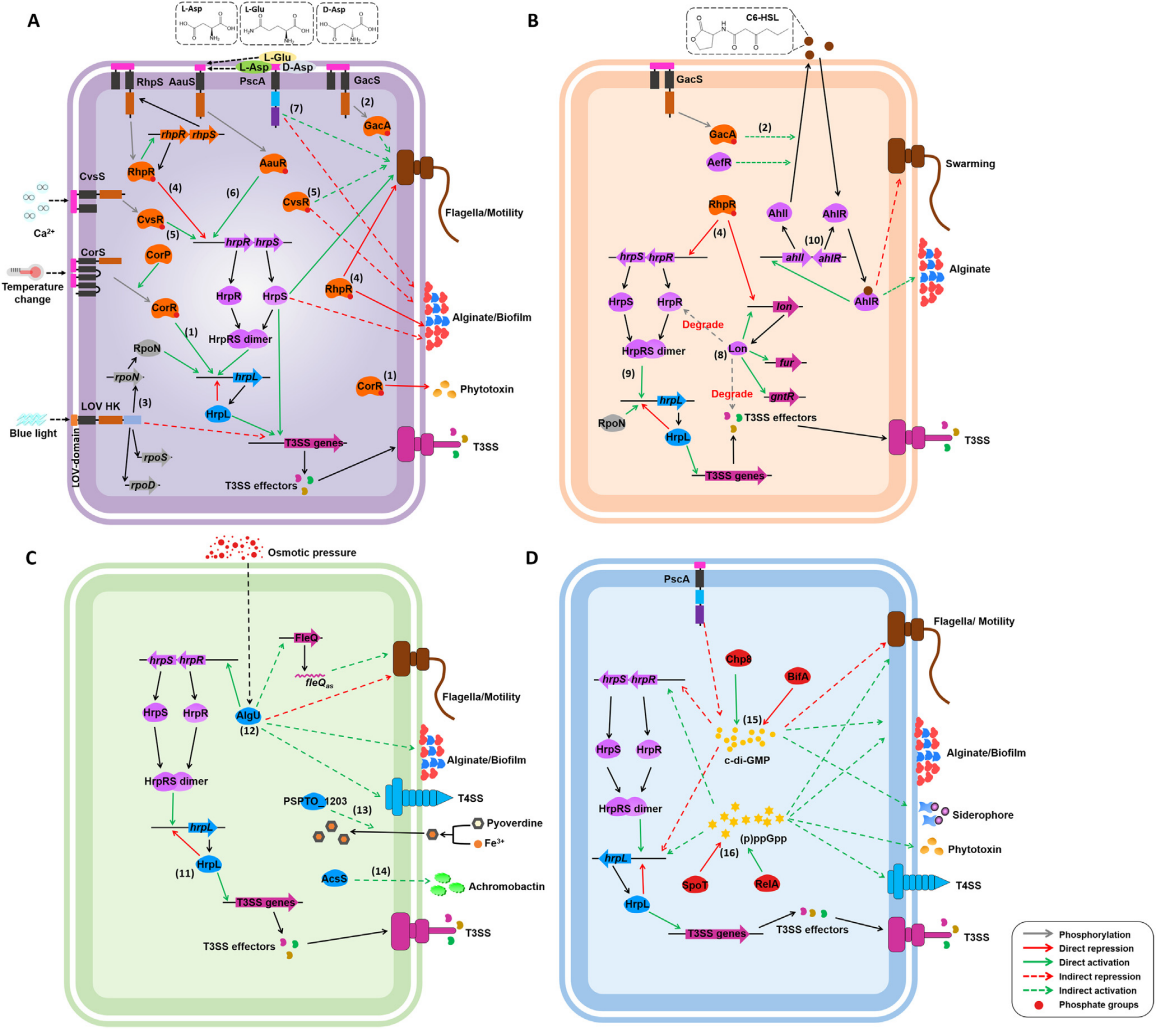
Model of signal transduction systems regulating behaviors in *P. syringae*. The numbers indicate the major signaling process. Black dash lines indicate the perceiving mechanisms are unknown. (A) TCSs and MCPs. (1) CorS is assumed to respond to temperature fluctuation through autophosphorylation, and further recruit CorR to regulate COR production. CorP is required for CorR activation. (2) GacAS is involved in the biosynthesis of C6-HSL and swarming. (3) Upon sensing blue light, LOV-HK hinders transcription of alternative sigma factor genes as well as T3SS genes. (4) After phosphorylated by RhpS or acetyl phosphate, RhpR suppresses the expression of *hrpRS* and *lon*, and inhibits swimming and biofilm production. (5) By sensing Ca^2+^ in environment, CvsRS activates transcription of *hrpRS* and *algU.* CvsR also inhibits bacterial cell attachment but contributes to swimming and swarming motility. (6) AauRS activates the transcription of *hrpRS* by sensing acidic amino acid signals in host cells. (7) Upon direct sensing l/d-Asp or l-Glu, PscA controls swarming motility, biofilm formation, and c-di-GMP production *in vivo.* (B) Lon, HrpRS and QS. (8) Lon protease degrades T3SS activator HrpR and a cluster of T3SS effectors. As transcriptional regulator, Lon suppresses its own expression and several metabolism pathways. (9) HrpR and HrpS form a heterodimer to directly activates the transcription of *hrpL.* HrpS independently regulates T3SS, motility and biofilm formation. (10) AhlI synthesizes C6-HSL, which works as AHL signal and then forms a stable complex with AhlR to activate the expression of *ahlI*, thus produces higher concentration of AHL with increasing bacterial populations. AhlI-AhlR QS system is further independently activated by AefR and GacA. (C) Extracytoplasmic function sigma factors. (11) HrpL directly activates most of T3SS genes and imposes spontaneous negative regulation of its own expression. (12) AlgU suppresses alginate biosynthesis, type VI secretion and motility through responding to environmental stress. (13) PSPTO_1203 controls pyoverdine uptake. (14) AcsS regulates the production and secretion of achromobactin. (D) Nucleotide-based secondary messengers. (15) Chp8 synthesizes c-di-GMP while BifA degrades c-di-GMP. c-di-GMP controls mRNA level of genes involved in flagellar assembly, exopolysaccharide biosynthesis, siderophore biosynthesis and T3SS. (16) The RelA protein generates (p)ppGpp from GTP and ATP, whereas SpoT is a bifunctional protein that synthesizes and hydrolyzes (p)ppGpp by sensing certain environmental cues. (p)ppGpp regulates multiple processes including T3SS, swarming motility, VI secretion system, exopolysaccharides and phytotoxin production. (For interpretation of the references to colour in this figure legend, the reader is referred to the web version of this article.)

Several *P. syringae* pathovars synthesise an endogenous phytotoxin named coronatine (COR) to facilitate stomatal re-opening when infecting host plants [23], [24]. In the *P. syringae* pv. *glycinea* PG4180 strain, the proteins involved in COR synthesis are encoded by a 90-kb plasmid at the virulence-promoting temperature (18 °C), but with a negligible level at its optimal growth temperature (28 °C) [25], [26]. The thermoregulation of COR production is modulated by an unconventional TCS consisting of three proteins: a membrane-embedded HK CorS protein, the RR CorR and an additional CorP protein (Fig. 1A, process 1) [27], [28]. CorS is assumed to respond to temperature fluctuations through a modulation of autophosphorylation, and to further transphosphorylate its cognate RR CorR to exert regulatory functions [29], [30]. CorS contains six transmembrane domains and potentially modifies its conformation by sensing the environmental temperature [29], [31]. Upon receiving a phosphate group from CorS, the phosphorylated CorR binds tightly to its target DNA in a thermo-responsive manner, thus activating COR biosynthesis [25], [27], [30], [31]. CorR is also reported to directly upregulate the expression of *hrpL* [32]. CorP is required for CorR activation, despite lacking a helix-turn-helix motif with which to bind DNA [25], [30]. However, the *Pto*DC3000 strain shows no temperature response in COR synthesis, and generates considerably less COR than the PG4180 strain [33], [34]*.*

### 2.2 Virulence regulatory TCS GacAS

GacAS, the first studied TCS, regulates the expression of virulence factors of *P. syringae* and is highly conserved as a global regulatory TCS of divergent cellular functions in many bacterial species [35]. In the *Pto*DC3000 strain, transposon insertion in *gacA* attenuates the induction of three central T3SS activators (*hrpR*, *hrpS* and *hrpL*), resulting in compromised bacterial virulence [36]. In addition, GacA is involved in the biosynthesis of N-acyl homoserine lactone (a compound involved in quorum sensing), pigment production and swarming, which are important for bacterial infection (Fig. 1A, process 2) [36], [37]. However, two recent studies propose the contrary view that GacAS negatively regulates the expression of T3SS genes (such as *avrPto* and *hrpL*) when invading a host plant [38], [39]. A newly constructed Δ*gacA*‐1 mutant strain generated using allelic exchange shows that GacA is indispensable for inoculated leaf infection by the *Pto*DC3000 strain, but is not required for apoplast colonisation in *Arabidopsis* leaf tissue [38]. A further study illustrates that the decreased virulence of a Tn5::*gacA* mutant strain is caused by a polar effect of *uvrC* and a nonsense mutation in *anmK* [39]. This revised model proposes that GacAS is triggered when infecting the leaf surface, but is deactivated during apoplast colonisation, thus working as a switch to exquisitely regulate motility and T3SS to escape the surveillance of host immunity [38], [39]. Our results in *P. savastanoi* pv. *phaseolicola* 1448A strain (*Psph*1448A, formerly known as *P. syringae* pv. *phaseolicola* 1448A) showed that GacA tends to negatively regulate *hrpRS* expression, but this may result from the differences between two strains.

### 2.3 Photosensory LOV-HK

Light is an important environmental cue in habitats and is sensed by multiple photosensory proteins in prokaryotes [40]. The genome sequence of the *P. syringae* pv. *syringae* B728a strain (*Pss*B728a) indicates the presence of three photosensory proteins, including two bacteriophytochromes (BphP1 and BphP2, both containing HK domain) and an HK-containing LOV (light, oxygen or voltage) domain (abbreviated as LOV-HK) [41], [42]. In the *Pss*B728a strain, the BphP1 bacteriophytochromes and LOV-HK cooperatively modulate swarming ability, whereas BphP2 has no significant influence on swarming [43]. When stimulated by either red or blue light, BphP1 suppresses bacterial swarming; however, the BphP1-controlled blue light signalling pathway is further repressed by the presence of LOV-HK [43]. Moreover, BphP1 contributes to leaf colonisation and lesion formation in bean pods (*Phaseolus vulgaris*) in a light-dependent manner [44]. In the *Pto*DC3000 strain, PSPTO_2896 encodes a hybrid protein (named Pst-Lov) containing an LOV domain that senses blue light, an HK autokinase domain and a receiver domain [45]. Pst-Lov hinders bacterial growth and significantly reduces the transcription of alternative sigma factor genes (*rpoN*, *rpoS* and *rpoD*), as well as T3SS genes (*hrpE*, *hopAA1-1*, *hrpL* and *hopL1*) in a particular light-dependent manner (Fig. 1A, process 3) [46]. Most importantly, Pst-Lov impedes the establishment of a *Pto*DC3000 infection in *Arabidopsis* leaves exposed to light, thus further attenuating the virulence of *P.* *syringae* against host plants [46]. Therefore, it is proposed that Pst-Lov functions as the ‘eyes’ of *P.* *syringae* to discriminate root and leaf habitats, thus reducing damage to the leaf tissue and buying time for dispersal to new hosts [46], [47], [48].

### 2.4 Master T3SS regulatory TCS RhpRS

RhpRS is one of the best illustrated TCSs in *P. syringae*, especially regarding its effects on T3SS regulation [49], [50], [51], [52]. The *rhpS* and *rhpR* genes are organised in one operon, where the *rhpS* gene encodes an HK and the *rhpR* gene encodes the cognate RR [50]. The *rhpS* mutant strain not only displays reduced expression of T3SS genes (such as *hrpR*, *hrpL* and *avrPto*), but also severely compromises pathogenicity in host plants [50]. RhpS is an autokinase and exerts kinase and phosphatase activity on RhpR [51]. The preferential roles of RhpS are environment-dependent, but the specific signal that this protein senses remains elusive. In nutrient-rich conditions, RhpR is phosphorylated by RhpS, then directly represses the *hrpRS*-*hrpL*-T3SS cascade (Fig. 1A, process 4) [49], [51]. However, when cultured in T3SS-inducing conditions, RhpS functions as a phosphatase to maintain RhpR proteins in low-phosphorylated states, thus allowing the induction of T3SS [51].

The small molecule acetyl phosphate is an intermediate in the phosphate acetyltransferase–acetate kinase pathway, and phosphorylates response regulators via direct phosphoryl transfer [53], [54], [55], [56], [57]. The purified recombinant RhpR protein can be phosphorylated by acetyl phosphate, and then induces the promoter binding affinity of RhpR [51]. Adding acetate to the culture medium induces the expression of *rhpR* in the *rhpS* mutant, but not in an *rhpRS* double mutant or the wild-type strain, indicating that acetyl phosphate acts as a phosphodonor to RhpR *in vivo* [51]. Acetyl phosphate potentially acts as an additional intracellular signal perceived by the RhpRS TCS to reflect the metabolic state of acetyl-CoA *in vivo* [57]. The phosphorylated state of RhpR protein at the conserved Asp70 (D70) site is required for its direct activation of the *rhpRS* operon by targeting an inverted repeat element (GTATC-N_6_-GATAC) in its own promoter [50], [52]. Overexpression of *rhpR* (D70A), a phosphorylation-defective mutant, in the *rhpRS* mutant background causes similar disease symptoms as the wild-type or *rhpRS* mutant strain, suggesting the important role of phosphorylated RhpR during bacterial infection [50].

Recent studies using both genome-wide chromatin immunoprecipitation sequencing (ChIP-seq) and transcriptome sequencing (RNA-seq) have provided evidences that the phosphorylation level of RhpR and the external surroundings significantly alter the regulatory roles of RhpRS in *P. syringae* [49], [51]. The phosphorylated RhpR is essential for the activation of T3SS genes (such as *hrpRS* and *hopR1*) and several other virulence-related phenotypes, including twitching motility, cyclic diguanylate (c-di-GMP) level, swimming motility, lipopolysaccharide production and biofilm formation [49]. When cultured in KB medium, RhpR directly regulates alcohol dehydrogenase activity, anthranilate synthase activity, cytochrome *c*_550_ accumulation and protease production, despite the strong inhibition of the pathogenicity associated with T3SS [49]. In conclusion, environmental signals and the phosphorylation state determine the regulatory preference of RhpRS in its function of regulating virulence and metabolism.

### 2.5 Metal ion sensing TCS CvsRS

Minerals are important signalling molecules and raw materials for bacteria [58]. For instance, Ca^2+^ is abundant in the leaf apoplast and acts as an important signalling molecule for phytopathogenic bacteria [59]. In the *Pto*DC3000 strain, CvsRS is a Ca^2+^-induced TCS composed of the HK CvsS and the RR CvsR [60]. CvsRS affects bacterial virulence by at least four means: 1) directly activating transcription of the *hrpRS* operon, thus upregulating T3SS induction; 2) indirectly suppressing expression of the ECF sigma factor AlgU and alginate production; 3) inhibiting bacterial cell attachment and 4) contributing to swimming and swarming motility (Fig. 1A, process 5) [60]. In addition, CvsRS modulates the expression of various metabolism-related genes, including the beta-carbonic anhydrase gene PSPTO_5255 and the putative sulphate permease PSPTO_5256, which suppress calcium precipitation [61]. However, the induction of *cvsRS* by Ca^2+^ is counteracted by the supplementation of glucose through an unknown mechanism [61].

### 2.6 Aspartate- and glutamate-sensing TCS AauRS

In the presence of sugars, a cluster of amino acids from plant extracts are capable of inducing the expression of T3SS genes in the *Pto*DC3000 strain [62]. A recent Tn*5* transposon mutagenesis screening study identifies that the TCS AauRS (encoded by the *amino acid uptake* locus) and the substrate-binding protein AatJ together activate the transcription of T3SS genes by sensing acidic amino acid signals in host cells [63]. AauRS activates the transcription of the adjacent *aatJQMP* operon in the presence of external acidic amino acid signals [63]. Among 15 amino acids tested in ligand profile, l-Asp and l-Glu show the highest levels of induction of the *aatJ* promoter [63]. Interestingly, l-Asp and l-Glu are the ligands of MCP PSPTO_2480 in *Pto*DC3000, which assist *Pto*DC3000 to invade apoplasts of tomato leaves (see details in Section 5) [64]. This implies that natural acidic amino acids in host plants not only help *P. syringae* to enter the plant tissue and colonize the intercellular apoplast space, but also further activate the T3SS expression. The response regulator AauR binds to an AauR-binding motif (TTCGG-N_4_-CCGAA) in the promoter of the *hrpRS* operon, thus directly activating its transcription and promoting bacterial virulence in *Arabidopsis* (Fig. 1A, process 6) [63]. The AauR-binding motif is highly conserved in the *hrpRS* promoter sequences among 17 bacterial strains with a canonical T3SS, indicating that this activation function is ancient [63]. Similar AauR regulation of *hrpRS* transcription and virulence has also been shown in the *Pss*B728a strain [63].

## 3 Extracytoplasmic function sigma factors

Bacteria contain two sigma factor families, σ^54^ and σ^70^. The largest and most diverse proteins in the σ^70^ family, the ECF sigma factors, enable bacteria to adapt to diverse environmental stimuli [65]. These specialised proteins are activated to alter bacterial responses to fluctuating environmental conditions, whereas in the absence of stimuli their activity is typically abolished by direct binding to a cytoplasmic membrane-bound anti-sigma factor protein [66], [67]. The genomic analysis of three sequenced *P. syringae* pathovars reveals 10 ECF sigma factors [68]. Half of these control the expression of genes involved in the iron homeostasis pathway (and are thus termed iron starvation sigma factors), while the other five are identified as stress response proteins [69]. Of the 10 factors, HrpL, AlgU and iron starvation sigma factors are reviewed in this section.

### 3.1 Master T3SS regulator HrpL

The ECF sigma factor HrpL regulates the virulence of *P. syringae* by modulating the expression of most T3SS genes [70], [71], [72]. Indeed, an *hrpL* mutant strain is unable to cause pathogenic symptoms in plants [73]. The induction of *hrpL* is directly activated by a transcription complex formed by HrpR, HrpS and the alternative sigma factor RpoN, but directly suppressed by HrpL itself [74], [75], [76], [77]. The self-negative regulation of *hrpL* allows the establishment of a balance between the invasion of plants to obtain nutrients and the evasion of the host immune system, thereby ensuring the survival and spread of bacteria. *hrpL* shares an intergenic upstream regulatory region with the *hrpJ* gene. The HrpRS-binding motif and host factor recognition elements in the *hrpL* promoter are occupied by a complex assembled by RNA polymerase and HrpL, resulting in the spontaneous negative regulation of *hrpL* (Fig. 1C, process 11) [77]. More details on the indirect and direct regulation of *hrpL* have been reviewed recently [78]. The multi-layered regulatory mechanisms of *hrpL* further show that *P. syringae* can finely regulate pathogenicity through sensing external environmental signals.

### 3.2 Global virulence regulator AlgU

Unlike HrpL, another ECF sigma factor, AlgU (synonyms, AlgT, RpoE, σ^22^), regulates multiple *P. syringae* virulence-related pathways, especially alginate biosynthesis and motility, by responding to environmental stress [79], [80], [81]. However, the role of AlgU differs among *P. syringae* pathovars. For instance, in the *Pss*B728a strain, the transcription of *algU* is activated by changes in external osmotic pressure, thus regulating the expression of genes involved in alginate biosynthesis, type VI secretion and oxidative stress responses (Fig. 1C, process 12) [82]. Besides osmotic and oxidative stress, in the *Pto*DC3000 strain, AlgU is involved in alginate production, flagellar biosynthesis and T3SS, and is thus a global regulator of pathogenic processes [81], [83]. During the establishment of *in planta Pto*DC3000 infection, AlgU negatively regulates the production of bacterial flagellin, a powerful inducer of the host immune response, to escape the surveillance of the plant immune system [81]. In the *Pto*DC3000 strain, AlgU also activates the antisense transcript of *fleQ* (*fleQ_as_*), which has a positive influence on flagellar motility [84]. Despite the diversity of its roles in different *P. syringae* pathovars, AlgU generally helps pathogenic bacteria to adapt to the environment and establish interactions with their host plants.

### 3.3 Iron starvation sigma factors

The ECF sigma factors that regulate downstream gene expression in response to siderophore binding are known as iron starvation (IS) sigma factors [85]. Five putative IS sigma factor genes have been identified in the genome of the *Pto*DC3000 strain: *pvdS*, PSPTO_0444, PSPTO_1203, PSPTO_1209 and PSPTO_1286 [69]. Externally provided iron ions regulate the transcription of *pvdS*, PSPTO_1209 and PSPTO_1286 [86]. A ChIP-seq analysis shows that PSPTO_1203 controls genes involved in pyoverdine uptake and production, while a promoter trap library screening study for PvdS reveals that it modulates the expression of genes by sensing hydroxamate siderophores (Fig. 1C, process 13) [87], [88]. In the *Pss*B728a strain, the IS sigma factor AcsS (Psyr_2580) functions as a regulator of the production and secretion of a newly discovered citrate siderophore, achromobactin (Fig. 1D, process 14) [89].

## 4 Nucleotide-based secondary messengers

Phytopathogens rely on intracellular secondary messengers to precisely sense external signals and rapidly control various cellular processes, including survival and pathogenesis [90], [91], [92]. In bacteria, in addition to the previously mentioned c-di-GMP, the nucleotide-based secondary messengers also include cyclic guanosine monophosphate (cGMP), cyclic adenosine monophosphate (cAMP), cyclic dimeric adenosine monophosphate (c-di-AMP) and guanosine tetra/penta-phosphate [(p)ppGpp] [92], [93], [94], [95]. In *P. syringae* pathovars, c-di-GMP and (p)ppGpp are the best studied secondary messengers.

### 4.1 c-di-GMP

Among bacteria, the production of c-di-GMP is mediated by two groups of highly conserved enzymes, diguanylate cyclases (DGCs) and phosphodiesterases (PDEs) [91], [96]. In the *Pto*DC3000 strain, the HrpRS-induced *chp8* gene encodes a DGC protein [97]. Chp8 dampens the production of flagellin but upregulates exopolysaccharide biosynthesis, thus promoting bacterial pathogenicity [97]. BifA is a PDE that degrades c-di-GMP and contributes to flagellar motility and virulence in the *Pto*DC3000 strain [98]. Transcriptome profiling has revealed that by overexpressing the *yedQ* gene (encoding the DGC of *Escherichia coli*) and the *yhjH* gene (encoding the PDE of *E. coli*) in the *Pss*B728a strain, c-di-GMP controls the mRNA levels of genes involved in flagellar assembly, exopolysaccharide biosynthesis, siderophore biosynthesis and oxidative stress resistance (Fig. 1D, process 15) [99]. Notably, a high c-di-GMP level significantly suppresses the induction of *hrpR*, *hrpL* and nine other T3SS effector genes, indicating that c-di-GMP potentially suppresses T3SS induction [99]. In addition, the promoter regions of three genes (Psyr_0610, Psyr_0685 and Psyr_5026) have been identified as c-di-GMP-responsive elements, and can be further used for reporter-based real-time measurements of c-di-GMP levels in *P. syringae* [99].

### 4.2 (p)ppGpp

(p)ppGpp is produced as a signalling compound in response to nutrient starvation, such as the shortage of carbon sources, fatty acids, phosphorus or iron [92], [100]. In the *Pss*B728a strain, the cellular concentration of (p)ppGpp is governed by two homologous enzymes, RelA and SpoT [101]. RelA protein generates (p)ppGpp from GTP and ATP, whereas SpoT is a bifunctional protein that synthesises and hydrolyses (p)ppGpp by sensing environmental cues [101]. In the *Pto*DC3000 strain, (p)ppGpp regulates multiple processes associated with virulence and survival, including T3SS, swarming motility, pyoverdine production, stress resistance and cell size (Fig. 1D, process 16) [101], [102]. A recent transcriptomic analysis identified the global effects of (p)ppGpp in both the *Pto*DC3000 and *Pss*B728a strains [103]. Generally, (p)ppGpp suppresses basic physical processes (such as nucleotide/amino acid/fatty acid metabolism), but activates virulence-related pathways (such as the type VI secretion system, exopolysaccharides and phytotoxin production) [103].

## 5 Methyl-accepting chemotaxis sensor proteins

Methyl-accepting chemotaxis sensor proteins (also known as chemoreceptors or MCPs) are the core of chemosensory pathways and have been found to assist plant pathogens in host invasion through stomata and wounds [104], [105]. The genome of the *Pto*DC3000 strain contains 49 putative MCP-coding genes, 36 of which possess the canonical topology characterized by a periplasmic ligand binding domain (LBD) flanked by two transmembrane regions [64]. Nine MCPs exhibit PAS (Per/ARNT/Sim) domains that are expected to be responsible for sensing certain intracellular signals [106]. The remaining four MCPs lacking the LBD have been proposed to sense physicochemical stimuli (such as osmotic stress or temperature) [64]. Although the chemotactic responses towards several attractants have been illustrated for *P. syringae* pathovars, the direct interactions between particular MCPs and hosts have been seldomly reported until recently [64], [107], [108]. By using thermal shift assays, l/d-Asp and l-Glu are recognised as signals of PSPTO_2480, a homologue of the amino acid receptor PscA [64]. Once perceiving its ligands, PSPTO_2480 mediates chemotaxis to the ligands recognized. In addition, mutation of the chemoreceptor gene alters swarming, biofilm and c-di-GMP levels (Fig. 1A, process 7) [64]. A crosstalk between the chemotaxis chemosensory pathway and other chemosensory pathways in *P. syringae* is hypothesized to be the base for these multiple responses. Because Asp and Glu are abundant in tomato apoplast, it is assumed that PSPTO_2480 specifically senses these amino acid ligands and assists *Pto*DC3000 to invade host plants [64].

Since plant infection with *Pto*DC3000 in the presence of d-Asp (the only non-metabolizable PSPTO_2480 ligand) significantly reduces virulence symptoms and the bacterial load inside the leaf, d-Asp can be used to reduce virulence of *Pto*DC3000 [64]. It is generally accepted that the crucial role of chemotaxis in plant infection is due to the chemotaxis towards compounds released from a wound or stomata [109]. In the presence of saturating ligand concentrations, there is no compound gradient towards the stomata preventing chemotaxis and efficient leaf entry. Study on PSPTO_2480 is a nice example of how interfering with signal transductions systems, by applying a key signal compound, can be used to reduce virulence.

## 6 Other intracellular surveillance systems

Other intracellular sensory systems are involved primarily in sensing abiotic and biotic inputs, thus contributing to the regulation of multiple key pathways [110]. Among these sensory systems, Lon, HrpS and quorum-sensing components are highlighted in this section.

### 6.1 Dual-function protein Lon

Lon, an ATP-dependent protease, is widely distributed in bacteria as well as eukaryotes [111]. In *P. syringae*, the Lon protease comprises both a C-terminal proteolytic domain and DNA-binding motif, implying its roles as both transcriptional regulator and protease. In KB medium, the transcription of *lon* is self-activated but is directly suppressed by phosphorylated RhpR (Fig. 1B, process 4) [51], [112]. Lon protease degrades the T3SS activator HrpR and a cluster of T3SS effectors (such as AvrPto, HopPtoM and HopPsyA), thus functioning as a T3SS repressor (Fig. 1B, process 8) [113], [114], [115]. A microarray analysis reveals that Lon inhibits the expression of T3SS genes and metabolic genes in KB but upregulates HrpL-regulated genes in MM, indicating that Lon is not only a dual-function protein, but also an environment sensory protein [116]. Although the molecular mechanism how Lon senses environmental stimuli is not clear, Lon is regulated by signal transduction pathways including RhpRS. Notably, further deletion of the *lon* gene in the *rhpS* mutant background results in a similar T3SS gene expression level and virulence to that of the wild-type strain, implying that Lon is a suppressor of the *rhpS* mutant [112]. In MM, the *lon* mRNA level is induced in several T3SS-deficient mutants, suggesting that the transcription of *lon* is inhibited by T3SS proteins via negative feedback [112].

By using multi-omic approaches, including ChIP-seq, RNA-seq and liquid chromatography-tandem mass spectrometry, our recent study demonstrates the different roles of Lon in response to different external signals [117]. As a DNA-binding transcriptional regulator, Lon directly mediates several metabolic pathways, including 1-dodecanol oxidation, glucokinase activity and pyoverdine production [117]. When acting as a protease, Lon proteolyses a group of T3SS effectors (including AvrB2, HrpW1 and HrcV) in KB but degrades metabolic factors (including NuoI and NoxB) in MM, suggesting that its protease activity depends on the extracellular environment [117].

### 6.2 Enhancer-binding proteins HrpR and HrpS

In *P. syringae*, both the HrpR and HrpS proteins belong to the family of enhancer-binding proteins (EBPs) that initiate gene transcription by utilising the alternative sigma factor σ^54^ [118]. Under T3SS-inducing conditions and with the help of the σ^54^ factor RpoN, a heterodimer is formed by HrpR and HrpS to directly activate the transcription of *hrpL* (Fig. 1B, process 9) [74]. The expression of the *hrpRS* operon and the assembly of the HrpRS heterodimer are strictly controlled by multiple factors. At least four TCSs (RhpRS, CvsRS, AauRS and GacAS), together with AlgU and HrpA, co-regulate the mRNA levels of *hrpRS* [37], [49], [51], [60], [63], [83]. Meanwhile, the formation of the HrpRS heterodimer is further controlled by the Lon protease and HrpGVFJ regulatory pathway at a post-transcriptional level [78], [110], [114], [115], [119], [120], [121]. A small reception domain composed of 12 residues has been identified at the N-terminal of the HrpS protein, indicating its potential role in sensing and responding to chemical and metabolic changes [118]. In the *Psph*1448A strain, HrpS alone not only activates the expression of various T3SS genes (such as *hrpK1*, *hrpA2* and *hopAJ1*) but also mediates a number of non-T3SS genes (such as PSPPH_1496, PSPPH_3494 and PSPPH_1525) [122]. Motility and biofilm formation are also regulated by the HrpS protein [122]. A recent study showes that HrpS protein is directly modified by an *Arabidopsis* metabolite, sulphoraphane, on the Cys209 site, leading to the suppression of T3SS and bacterial virulence [103].

### 6.3 One component system AhlR

One component systems (OCSs) are transcriptional regulators that respond to extracytosolic signals that are either taken up by the bacterium or diffuse across the membrane [123]. A representative example is the mechanism of quorum sensing (QS). QS is an exquisite process by which bacteria gauge their population size and coordinate their gene expression by perceiving small signalling molecules secreted by conspecific cells [124], [125]. In numerous Gram-negative pathogenic bacteria, including *P. syringae*, N-acyl homoserine lactone (AHL) is the QS signal molecule that indicates successful invasion of the host. However, AHL‐mediated QS has been less studied in *P. syringae* than in other bacteria such as *P. aeruginosa*. In the *Pss*B728a strain, the production of AHL is directly regulated by both the AhlI synthase and the regulatory protein AhlR [126], [127]. AhlI and AhlR are LuxI/R quorum regulator homologs. In the presence of metabolite precursors, AhlI synthesises 3-oxo-hexanoyl-homoserine lactone (3-oxo-C6-HSL), which acts as an AHL signal and forms a stable complex with AhlR to activate the expression of *ahlI*, thus producing higher concentrations of AHL with increasing bacterial populations (Fig. 1B, process 10) [126]. This AhlI–AhlR QS system is further independently activated by two other regulatory proteins: AefR and GacA (Fig. 1B, process 2) [36], [126], [127]. AefR is a TetR family transcriptional regulator that inhibits siderophore production, tolerance to antibiotics and its own expression, but activates T3SS by inducing *hrpL* [128], [129]. AhlI–AhlR QS mediates several phenotypes in *P. syringae*, including the positive regulation of exopolysaccharide production, oxidative stress tolerance and epiphytic fitness *in planta* and negative regulation of swarming motility [130].

## 7 Conclusions and perspectives

A successful plant pathogen must use elaborate signalling networks with both abiotic and biotic inputs to perceive and rapidly respond to its external environments. Evidently, *P. syringae* pathovars have evolved exquisite mechanisms to regulate their pathogenicity and metabolic pathways by recognising host plants or plant-associated environmental factors, thereby effectively avoiding the surveillance of the host immune system. Such capabilities have contributed to the large-scale transmission of these pathovars in farmland. In recent years, tremendous progress has been achieved in deciphering how *P. syringae* responds to different cues during plant–microbe interactions, thus uncovering its strict sensory logics. In the future, signal transduction systems are among the major targets for antimicrobial therapy. Traditional antibiotics that inhibit bacterial growth can generate an evolutionary pressure inducing the selection of resistant strains. In contract, designing target drugs to block key signal transduction pathways or interfere with the normal function of key proteins (such as TCSs or MCPs *etc.*) can disrupt bacterial functions without interfering with bacterial growth.

Various membrane-bound or intracellular signalling systems have been well characterised to participate in *P. syringae* signalling transduction networks (Table 1). Among these signalling schemes, TCSs have mostly been understudied. Given the important roles of TCSs in other pathogenic bacteria, an exploration of their functional mechanisms and signals is greatly significant to an understanding of the pathogenicity and metabolism of *P. syringae* under different environments. Over the past two decades, genomic analyses of *P. syringae* pathovars have identified most of the genes that encode proteins with signal-sensing domains. Nowadays, with the wide use of high-throughput sequencing technologies, it is becoming easier to decipher specific functions and mechanisms of these signal transduction schemes. Unveiling the genome-wide signal transduction network will not only provide a better understanding of bacterial preferences but will also contribute to the development of eco-friendly and sustainable methods to control the economic losses caused by *P. syringae* in the future.

**Table 1:** Overview of known signal transduction systems in *Pseudomonas syringae.*

Sensory components	Signal transduction categories	Signals/secondary messengers	Functions and mechanisms	References
CorRS	TCS	Temperature changes	CorR activates COR biosynthesis and *hrpL* expression.	[27], [28], [30], [32]

GacAS	TCS	Unknown	GacA regulates several virulence-related pathways, including AHL production, T3SS, and swarming.	[35], [36], [37], [38]

LOV-HK	TCS	Blue light	LOV-HK reduces transcription of several alternative sigma factor genes (*rpoN*, *rpoS* and *rpoD*), T3SS genes (*hrpE*, *hopAA1-1*, *hrpL* and *hopL1*), and modulate swarming motility.	[43], [46]

RhpRS	TCS	Unknown	Phosphorylated RhpR directly suppresses the expression of *hrpRS* and *lon*. RhpR regulates twitching motility, c-di-GMP level, swimming motility, lipopolysaccharide production and biofilm formation in a phosphorylation-dependent manner. In KB, RhpR regulates alcohol dehydrogenase activity, anthranilate synthase activity, cytochrome *c*_550_ accumulation and protease production.	[49], [51], [112]

CvsRS	TCS	Ca^2+^	CvsRS affects bacterial virulence and metabolism, including T3SS, alginate production, cell attachment, swimming and swarming motility.	[60], [61]

AauRS	TCS	Acidic amino acid	AauR directly activates the transcription of *hrpRS* and promotes bacterial virulence in *Arabidopsis*.	[63]

HrpL	ECF sigma factor	Unknown	HrpL directly activates the expression of most T3SS genes. HrpL also imposes spontaneous negative regulation of its own expression.	[75], [77]

AlgU	ECF sigma factor	External osmotic pressure	AlgU is involved in alginate production, flagella biosynthesis, T3SS, type VI secretion and oxidative stress responses.	[81], [82], [83], [84]

Iron starvation sigma factors	ECF sigma factor	Iron ions	PSPTO_1203 controls pyoverdine uptake. AcsS (Psyr_2580) regulates the production and secretion of achromobactin.	[88], [89]

Chp8	Nucleotide-based secondary messengers	c-di-GMP	Chp8 functions as diguanylate cyclase to synthesize c-di-GMP, and then inhibits the production of flagellin but upregulates exopolysaccharide biosynthesis. Synthesized c-di-GMP controls T3SS, flagellar assembly, exopolysaccharide biosynthesis, siderophore biosynthesis, and oxidative stress resistance.	[97], [99]

BifA	Nucleotide-based secondary messengers	c-di-GMP	BifA protein acts as a phosphodiesterase to degrade c-di-GMP *in vivo*.	[98]

RelA, SpoT	Nucleotide-based secondary messengers	(p)ppGpp	The RelA protein generates (p)ppGpp from GTP and ATP, whereas SpoT is a bifunctional protein that synthesizes and hydrolyzed (p)ppGpp. (p)ppGpp regulates multiple processes associated with virulence and survival including nucleotide/amino acid/ fatty acid metabolism, exopolysaccharides production, type VI secretion system, phytotoxin production, T3SS, swarming motility, pyoverdine production, stress resistance, and cell sizes.	[101], [102], [103]

PscA	MCP	Acidic amino acid	PscA controls swarming motility, biofilm formation and c-di-GMP production, and bacterial virulence.	[64]

Lon	Intracellular surveillance system	Unknown	Lon protease degrades T3SS activator HrpR and a cluster of T3SS effectors (such as AvrPto, HopPtoM and HopPsyA), thus functions as a T3SS repressor. As a DNA-binding transcriptional regulator, Lon directly mediates several metabolism pathways, including 1-dodecanol oxidation, glucokinase activity, and pyoverdine production. When acting as a protease, Lon cuts several T3SS effectors (including AvrB2, HrpW1 and HrcV) in KB but degrades metabolic factors (including NuoI and NoxB) in MM.	[113], [114], [115], [116], [117]

HrpR and HrpS	Intracellular surveillance system	Unknown	HrpR and HrpS form a heterodimer to directly activates the transcription of *hrpL*. HrpS alone regulates T3SS, motility and biofilm formation. Sulphoraphane directly modifies HrpS protein and suppresses T3SS and bacterial virulence.	[74], [103], [122]

AhlI-AhlR	Quorum sensing system/OCS	3-oxo-hexanoyl-homoserine lactone	AhlI synthesizes C6-HSL, which works as AHL signal and then forms a stable complex with AhlR to activate the expression of *ahlI*. AhlI-AhlR system is independently activated by AefR and GacA.	[36], [126], [127]

## Declaration of Competing Interest

The authors declare that they have no known competing financial interests or personal relationships that could have appeared to influence the work reported in this paper.

## References

[1] Bliska J.B. (1993). Signal transduction in the mammalian cell during bacterial attachment and entry. Cell.

[2] Hazelbauer G.L. (1993). Bacterial motility and signal transduction. Cell.

[3] Stock J.B. (1990). Signal transduction in bacteria. Nature.

[4] Stock J. (1994). Chemosensing and signal transduction in bacteria. Curr Opin Neurobiol.

[5] Hellingwerf K.J. (1995). Signal transduction in bacteria: phospho-neural network(s) in *Escherichia coli*?. FEMS Microbiol Rev.

[6] Parkinson J.S. (1993). Signal transduction schemes of bacteria. Cell.

[7] Hengge R. (2009). Principles of c-di-GMP signalling in bacteria. Nat Rev Microbiol.

[8] Galperin M.Y. (2005). A census of membrane-bound and intracellular signal transduction proteins in bacteria: bacterial IQ, extroverts and introverts. BMC Microbiol.

[9] Galperin M.Y. (2004). Bacterial signal transduction network in a genomic perspective. Environ Microbiol.

[10] Galperin M.Y. (2018). What bacteria want. Environ Microbiol.

[11] Preston G.M. (2000). *Pseudomonas syringae* pv. *tomato*: the right pathogen, of the right plant, at the right time. Mol. Plant Pathol.

[12] Mansfield J. (2012). Top 10 plant pathogenic bacteria in molecular plant pathology. Mol Plant Pathol.

[13] Xin X.-F. (2018). *Pseudomonas syringae*: what it takes to be a pathogen. Nat Rev Microbiol.

[14] Galan J.E., Collmer A. (1999). Type III secretion machines: bacterial devices for protein delivery into host cells. Science.

[15] Cunnac S. (2009). *Pseudomonas syringae* type III secretion system effectors: repertoires in search of functions. Curr Opin Microbiol.

[16] Huynh T.V. (1989). Bacterial blight of soybean: regulation of a pathogen gene determining host cultivar specificity. Science.

[17] Rahme L.G. (1992). Plant and environmental sensory signals control the expression of hrp genes in *Pseudomonas syringae* pv. *phaseolicola*. J Bacteriol.

[18] Xiao Y. (1992). Organization and environmental regulation of the *Pseudomonas syringae* pv. *syringae* 61 *hrp* cluster. J Bacteriol.

[19] Ulrich, L.E. and Zhulin, I.B. (2010) The MiST2 database: a comprehensive genomics resource on microbial signal transduction. Nucleic Acids Res, 38 (Database issue), D401-7. 10.1093/nar/gkp940 PMC2808908 19900966

[20] Mascher T. (2006). Stimulus perception in bacterial signal-transducing histidine kinases. Microbiol Mol Biol Rev.

[21] Stock A.M. (2000). Two-component signal transduction. Annu Rev Biochem.

[22] Zschiedrich C.P. (2016). Molecular Mechanisms of Two-Component Signal Transduction. J Mol Biol.

[23] Bender C.L. (1999). *Pseudomonas syringae* phytotoxins: mode of action, regulation, and biosynthesis by peptide and polyketide synthetases. Microbiol Mol Biol Rev.

[24] Panchal S. (2016). Coronatine facilitates *Pseudomonas syringae* infection of arabidopsis leaves at night. Front Plant Sci.

[25] Wang L. (1999). The transcriptional activator CorR is involved in biosynthesis of the phytotoxin coronatine and binds to the cmaABT promoter region in a temperature-dependent manner. Mol Gen Genet.

[26] Bender C.L. (1993). Characterization of the genes controlling the biosynthesis of the polyketide phytotoxin coronatine including conjugation between coronafacic and coronamic acid. Gene.

[27] Ullrich M. (1995). A modified two-component regulatory system is involved in temperature-dependent biosynthesis of the *Pseudomonas syringae* phytotoxin coronatine. J Bacteriol.

[28] Penaloza-Vazquez A., Bender C.L. (1998). Characterization of CorR, a transcriptional activator which is required for biosynthesis of the phytotoxin coronatine. J Bacteriol.

[29] Smirnova A.V., Ullrich M.S. (2004). Topological and deletion analysis of CorS, a *Pseudomonas syringae* sensor kinase. Microbiology.

[30] Rangaswamy V., Bender C.L. (2000). Phosphorylation of CorS and CorR, regulatory proteins that modulate production of the phytotoxin coronatine in *Pseudomonas syringae*. FEMS Microbiol Lett.

[31] Smirnova A.V. (2002). Control of temperature-responsive synthesis of the phytotoxin coronatine in *Pseudomonas syringae* by the unconventional two-component system CorRPS. J Mol Microbiol Biotechnol.

[32] Sreedharan A. (2006). CorR regulates multiple components of virulence in *Pseudomonas syringae* pv. *tomato* DC3000. Mol Plant Microbe Interact.

[33] Brooks D.M. (2004). Identification and characterization of a well-defined series of coronatine biosynthetic mutants of *Pseudomonas syringae* pv. *tomato* DC3000. Mol Plant Microbe Interact.

[34] Braun Y. (2008). Component and protein domain exchange analysis of a thermoresponsive, two-component regulatory system of *Pseudomonas syringae*. Microbiology.

[35] Heeb S., Haas D. (2001). Regulatory roles of the GacS/GacA two-component system in plant-associated and other gram-negative bacteria. Mol Plant Microbe Interact.

[36] Chatterjee A. (2003). GacA, the response regulator of a two-component system, acts as a master regulator in *Pseudomonas syringae* pv. *tomato* DC3000 by controlling regulatory RNA, transcriptional activators, and alternate sigma factors. Mol Plant Microbe Interact.

[37] Ortiz-Martin I. (2010). Positive regulation of the Hrp type III secretion system in *Pseudomonas syringae* pv. *phaseolicola*. Mol Plant Microbe Interact.

[38] O'Malley M.R. (2020). A revised model for the role of GacS/GacA in regulating type III secretion by *Pseudomonas syringae* pv. *tomato* DC3000. Mol. Plant Pathol.

[39] O'Malley M.R. (2019). Re-evaluation of a Tn5::gacA mutant of *Pseudomonas syringae* pv. *tomato* DC3000 uncovers roles for uvrC and anmK in promoting virulence. PLoS ONE.

[40] Kiang N.Y. (2007). Spectral signatures of photosynthesis. I. Review of Earth organisms. Astrobiology.

[41] Swartz T.E. (2007). Blue-light-activated histidine kinases: two-component sensors in bacteria. Science.

[42] Bhoo S.H. (2001). Bacteriophytochromes are photochromic histidine kinases using a biliverdin chromophore. Nature.

[43] Wang G.Y. (2013). Critical regulation of miR-200/ZEB2 pathway in Oct4/Sox2-induced mesenchymal-to-epithelial transition and induced pluripotent stem cell generation. Proc Natl Acad Sci U S A.

[44] McGrane, R, Beattie, GA (2017) Pseudomonas syringae pv. syringae B728a regulates multiple stages of plant colonization via the bacteriophytochrome BphP1. mBio 8 (5). 10.1128/mBio.01178-17 PMC5654926 29066541

[45] Cao Z. (2008). A blue light inducible two-component signal transduction system in the plant pathogen *Pseudomonas syringae* pv. *tomato*. Biophys J.

[46] Moriconi V. (2013). LOV-domain photoreceptor, encoded in a genomic island, attenuates the virulence of *Pseudomonas syringae* in light-exposed *Arabidopsis* leaves. Plant J.

[47] Jackson R.W. (2011). Bacterial pathogen evolution: breaking news. Trends Genet.

[48] Alizon S. (2009). Virulence evolution and the trade-off hypothesis: history, current state of affairs and the future. J Evol Biol.

[49] Xie Y. (2019). *Pseudomonas savastanoi* two-component system RhpRS Switches between virulence and metabolism by tuning phosphorylation state and sensing nutritional conditions. MBio.

[50] Xiao Y. (2007). Two-component sensor RhpS promotes induction of *Pseudomonas syringae* type III secretion system by repressing negative regulator RhpR. Mol Plant Microbe Interact.

[51] Deng X. (2014). Molecular mechanisms of two-component system RhpRS regulating type III secretion system in *Pseudomonas syringae*. Nucleic Acids Res.

[52] Deng X. (2010). *Pseudomonas syringae* two-component response regulator RhpR regulates promoters carrying an inverted repeat element. Mol Plant Microbe Interact.

[53] Klein A.H. (2007). The intracellular concentration of acetyl phosphate in *Escherichia coli* is sufficient for direct phosphorylation of two-component response regulators. J Bacteriol.

[54] Heyde M. (2000). Involvement of carbon source and acetyl phosphate in the external-pH-dependent expression of porin genes in *Escherichia coli*. J Bacteriol.

[55] Quon K.C. (1996). Cell cycle control by an essential bacterial two-component signal transduction protein. Cell.

[56] Xu H. (2010). Role of acetyl-phosphate in activation of the Rrp2-RpoN-RpoS pathway in *Borrelia burgdorferi*. PLoS Pathog.

[57] Wolfe A.J. (2005). The acetate switch. Microbiol Mol Biol Rev.

[58] Gadd G.M. (2010). Metals, minerals and microbes: geomicrobiology and bioremediation. Microbiology.

[59] Stael S. (2012). Plant organellar calcium signalling: an emerging field. J Exp Bot.

[60] Fishman M.R. (2018). Ca(2+)-induced two-component system CvsSR regulates the type III secretion system and the extracytoplasmic function sigma factor AlgU in *Pseudomonas syringae* pv. *tomato* DC3000. J Bacteriol.

[61] Fishman M.R., Filiatrault M.J. (2019). Prevention of surface-associated calcium phosphate by the *Pseudomonas syringae* two-component system CvsSR. J Bacteriol.

[62] Anderson J.C. (2014). Decreased abundance of type III secretion system-inducing signals in *Arabidopsis mkp1* enhances resistance against *Pseudomonas syringae*. Proc Natl Acad Sci U S A.

[63] Yan Q. (2020). Ancient co-option of an amino acid ABC transporter locus in *Pseudomonas syringae* for host signal-dependent virulence gene regulation. PLoS Pathog.

[64] Cerna-Vargas J.P. (2019). Chemoperception of Specific Amino Acids Controls Phytopathogenicity in *Pseudomonas syringae* pv. *tomato*. mBio.

[65] Osterberg S. (2011). Regulation of alternative sigma factor use. Annu Rev Microbiol.

[66] Gruber T.M., Gross C.A. (2003). Multiple sigma subunits and the partitioning of bacterial transcription space. Annu Rev Microbiol.

[67] Hughes K.T., Mathee K. (1998). The anti-sigma factors. Annu Rev Microbiol.

[68] Thakur P.B. (2013). Characterization of five ECF sigma factors in the genome of *Pseudomonas syringae* pv. *syringae* B728a. PLoS ONE.

[69] Oguiza J.A. (2005). Extracytoplasmic function sigma factors in *Pseudomonas syringae*. Trends Microbiol.

[70] Xiao Y. (1994). Identification of a putative alternate sigma factor and characterization of a multicomponent regulatory cascade controlling the expression of *Pseudomonas syringae* pv. *syringae* Pss61 *hrp* and hrmA genes. J Bacteriol.

[71] Lam H.N. (2014). Global analysis of the HrpL regulon in the plant pathogen *Pseudomonas syringae* pv. *tomato* DC3000 reveals new regulon members with diverse functions. PLoS ONE.

[72] Fouts D.E. (2002). Genomewide identification of *Pseudomonas syringae* pv. *tomato* DC3000 promoters controlled by the HrpL alternative sigma factor. Proc Natl Acad Sci U S A.

[73] Soylu S. (2005). Cellular reactions in *Arabidopsis* following challenge by strains of *Pseudomonas syringae*: from basal resistance to compatibility. Physiol Mol Plant Pathol.

[74] Hutcheson S.W. (2001). Enhancer-binding proteins HrpR and HrpS interact to regulate hrp-encoded type III protein secretion in *Pseudomonas syringae* strains. J Bacteriol.

[75] Xiao Y., Hutcheson S.W. (1994). A single promoter sequence recognized by a newly identified alternate sigma factor directs expression of pathogenicity and host range determinants in *Pseudomonas syringae*. J Bacteriol.

[76] Hendrickson E.L. (2000). The alternative sigma factor RpoN is required for hrp activity in *Pseudomonas syringae* pv. *maculicola* and acts at the level of hrpL transcription. J Bacteriol.

[77] Waite C. (2017). Negative autogenous control of the master type III secretion system regulator HrpL in *Pseudomonas syringae*. mBio.

[78] Xie Y. (2019). Regulation of type III secretion system in *Pseudomonas syringae*. Environ Microbiol.

[79] Keith L.M., Bender C.L. (2001). Genetic divergence in the algT-muc operon controlling alginate biosynthesis and response to environmental stress in *Pseudomonas syringae*. DNA Seq.

[80] Schenk A. (2008). The alternative sigma factor AlgT, but not alginate synthesis, promotes in planta multiplication of *Pseudomonas syringae* pv. *glycinea*. Microbiology 154 (Pt.

[81] Bao Z. (2020). *Pseudomonas syringae* AlgU downregulates flagellin gene expression. helping evade plant immunity. J. Bacteriol..

[82] Yu X. (2014). Transcriptional analysis of the global regulatory networks active in *Pseudomonas syringae* during leaf colonization. mBio.

[83] Markel E. (2016). AlgU controls expression of virulence genes in *Pseudomonas syringae* pv. *tomato* DC3000. J Bacteriol.

[84] Markel E. (2018). An AlgU-regulated antisense transcript encoded within the *Pseudomonas syringae* fleQ gene has a positive effect on motility. J Bacteriol.

[85] Braun V. (2006). Gene regulation by transmembrane signaling. Biometals.

[86] Bronstein P.A. (2008). Global transcriptional responses of *Pseudomonas syringae* DC3000 to changes in iron bioavailability in vitro. BMC Microbiol.

[87] Swingle B. (2008). Characterization of the PvdS-regulated promoter motif in *Pseudomonas syringae* pv. *tomato* DC3000 reveals regulon members and insights regarding PvdS function in other pseudomonads. Mol Microbiol.

[88] Markel E. (2011). An extracytoplasmic function sigma factor-mediated cell surface signaling system in *Pseudomonas syringae* pv. *tomato* DC3000 regulates gene expression in response to heterologous siderophores. J Bacteriol.

[89] Greenwald J.W. (2012). RNA-seq analysis reveals that an ECF sigma factor, AcsS, regulates achromobactin biosynthesis in *Pseudomonas syringae* pv. *syringae* B728a. PLoS ONE.

[90] Pesavento C., Hengge R. (2009). Bacterial nucleotide-based second messengers. Curr Opin Microbiol.

[91] Romling U. (2013). Cyclic di-GMP: the first 25 years of a universal bacterial second messenger. Microbiol Mol Biol Rev.

[92] Kalia D. (2013). Nucleotide, c-di-GMP, c-di-AMP, cGMP, cAMP, (p)ppGpp signaling in bacteria and implications in pathogenesis. Chem Soc Rev.

[93] Magnusson L.U. (2005). ppGpp: a global regulator in *Escherichia coli*. Trends Microbiol.

[94] Witte G. (2008). Structural biochemistry of a bacterial checkpoint protein reveals diadenylate cyclase activity regulated by DNA recombination intermediates. Mol Cell.

[95] Ross P. (1987). Regulation of cellulose synthesis in *Acetobacter xylinum* by cyclic diguanylic acid. Nature.

[96] Chan C. (2004). Structural basis of activity and allosteric control of diguanylate cyclase. Proc Natl Acad Sci U S A.

[97] Engl C. (2014). Chp8, a diguanylate cyclase from *Pseudomonas syringae* pv. *Tomato* DC3000, suppresses the pathogen-associated molecular pattern flagellin, increases extracellular polysaccharides, and promotes plant immune evasion. mBio.

[98] Aragon I.M. (2015). The c-di-GMP phosphodiesterase BifA is involved in the virulence of bacteria from the *Pseudomonas syringae* complex. Mol Plant Pathol.

[99] Wang T. (2019). Pleiotropic effects of c-di-GMP content in *Pseudomonas syringae*. Appl Environ Microbiol.

[100] Vercruysse M. (2011). Stress response regulators identified through genome-wide transcriptome analysis of the (p)ppGpp-dependent response in *Rhizobium etli*. Genome Biol.

[101] Chatnaparat T. (2015). The bacterial alarmone (p)ppGpp is required for virulence and controls cell size and survival of *Pseudomonas syringae* on plants. Environ Microbiol.

[102] Chatnaparat T. (2015). The Stringent Response Mediated by (p)ppGpp is required for virulence of *Pseudomonas syringae* pv. *tomato* and its survival on *Tomato*. Mol Plant Microbe Interact.

[103] Wang W. (2020). An *Arabidopsis* secondary metabolite directly targets expression of the bacterial type III secretion system to inhibit bacterial virulence. Cell Host Microbe.

[104] Matilla M.A., Krell T. (2018). The effect of bacterial chemotaxis on host infection and pathogenicity. FEMS Microbiol Rev.

[105] Antunez-Lamas M. (2009). Bacterial chemoattraction towards jasmonate plays a role in the entry of *Dickeya dadantii* through wounded tissues. Mol Microbiol.

[106] Henry J.T., Crosson S. (2011). Ligand-binding PAS domains in a genomic, cellular, and structural context. Annu Rev Microbiol.

[107] Kim H.E. (2007). Ethylene chemotaxis in *Pseudomonas aeruginosa* and other *Pseudomonas* species. Microbes Environ.

[108] Cuppels D.A. (1988). Chemotaxis by *Pseudomonas syringae* pv. *tomato*. Appl Environ Microbiol.

[109] Matilla M.A., Krell T. (2018). The effect of bacterial chemotaxis on host infection and pathogenicity. FEMS Microbiol Rev.

[110] Jovanovic M. (2011). Regulation of the co-evolved HrpR and HrpS AAA+ proteins required for *Pseudomonas syringae* pathogenicity. Nat Commun.

[111] Sauer R.T., Baker T.A. (2011). AAA+ proteases: ATP-fueled machines of protein destruction. Annu Rev Biochem.

[112] Zhou T. (2016). Lon protease is involved in RhpRS-mediated regulation of type III secretion in *Pseudomonas syringae*. Mol Plant Microbe Interact.

[113] Losada L.C., Hutcheson S.W. (2005). Type III secretion chaperones of *Pseudomonas syringae* protect effectors from Lon-associated degradation. Mol Microbiol.

[114] Bretz J. (2002). Lon protease functions as a negative regulator of type III protein secretion in *Pseudomonas syringae*. Mol Microbiol.

[115] Ortiz-Martin I. (2010). Negative regulation of the Hrp type III secretion system in *Pseudomonas syringae* pv. *phaseolicola*. Mol Plant Microbe Interact.

[116] Lan L. (2007). Mutation of Lon protease differentially affects the expression of *Pseudomonas syringae* type III secretion system genes in rich and minimal media and reduces pathogenicity. Mol Plant Microbe Interact.

[117] Hua C. (2020). *Pseudomonas syringae* dual-function protein Lon switches between virulence and metabolism by acting as both DNA-binding transcriptional regulator and protease in different environments. Environ Microbiol.

[118] Shingler V. (1996). Signal sensing by sigma 54-dependent regulators: derepression as a control mechanism. Mol Microbiol.

[119] Huang Y.C. (2016). The pathogenicity factor HrpF interacts with HrpA and HrpG to modulate type III secretion system (T3SS) function and t3ss expression in *Pseudomonas syringae* pv. *averrhoi*. Mol Plant Pathol.

[120] Wei C.F. (2005). A chaperone-like HrpG protein acts as a suppressor of HrpV in regulation of the *Pseudomonas syringae* pv. syringae type III secretion system. Mol Microbiol.

[121] Charova S.N. (2018). Migration of type III secretion system transcriptional regulators links gene expression to secretion. mBio.

[122] Wang, J. et al. (2018) HrpS is a global regulator on type III secretion system (T3SS) and non-T3SS genes in Pseudomonas savastanoi pv. phaseolicola. Mol Plant Microbe Interact, MPMI02180035R. 10.1094/MPMI-02-18-0035-R 29717915

[123] Ulrich L.E. (2005). One-component systems dominate signal transduction in prokaryotes. Trends Microbiol.

[124] Von Bodman S.B. (2003). Quorum sensing in plant-pathogenic bacteria. Annu Rev Phytopathol.

[125] Loh J. (2002). Quorum sensing in plant-associated bacteria. Curr Opin Plant Biol.

[126] Quinones B. (2004). Regulation of AHL production and its contribution to epiphytic fitness in *Pseudomonas syringae*. Mol Plant Microbe Interact.

[127] Dumenyo C.K. (1998). Genetic and physiological evidence for the production of N-acyl homoserine lactones by *Pseudomonas syringae* pv. *syringae* and other fluorescent plant pathogenic Pseudomonas species. Eur J Plant Pathol.

[128] Yun S. (2015). Functional analysis of the *aefR* mutation and identification of its binding site in *Pseudomonas syringae* pv. *tabaci* 11528. Acta Biochim Biophys Sin (Shanghai).

[129] Deng X. (2009). *Pseudomonas syringae* pv. *phaseolicola* Mutants Compromised for type III secretion system gene induction. Mol Plant Microbe Interact.

[130] Quinones B. (2005). Quorum sensing regulates exopolysaccharide production, motility, and virulence in *Pseudomonas syringae*. Mol Plant Microbe Interact.

